# Evidence-based Recommendations to Inform Best Practices for LGBTQ Older Adults in Long-term Care Settings

**DOI:** 10.1093/geroni/igab046.3089

**Published:** 2021-12-17

**Authors:** Katherine Fasullo, Erik McIntosh, Susan W. Buchholz, Todd Ruppar, Sarah Ailey

**Affiliations:** 1 Rush University, Chicago, Illinois, United States; 2 Michigan State University, East Lansing, Michigan, United States; 3 Rush University College of Nursing, Chicago, Illinois, United States

## Abstract

Lesbian, gay, bisexual, transgender, and queer (LGBTQ) older adults are more likely to live alone and have less familial support, which disproportionately contributes to a reliance on long-term care facilities as they age. Best-practice guidelines supported by scholarly literature to care for LGBTQ older adults in long-term care settings do not exist. This review synthesizes literature about LGBTQ older adults in long-term care facilities and provides recommendations for best practice guideline development. Four electronic databases were searched in June 2019 for studies conducted between 2000 – 2019 related to caring for LGBTQ older adults in long-term care settings. An integrative literature review was completed on the twenty eligible studies. Findings showed that LGBTQ participants fear discrimination in long-term care leading to the invisibility of their identities. They recognize a need for increased staff training and the importance of community networks and facility preferences. Long-term care staff have mixed experiences with inclusive practices and complex views of LGBTQ older adults. They experience training deficits and have a need for more expansive training modalities. The recommendations offered by both LGBTQ participants and long-term care staff are to revise policies and forms as well as provide widespread training and education. LGBTQ participants recommend that their unique identities be recognized within long-term care while long-term care staff recommend leadership involvement to change culture and practice. This review provides evidence-based recommendations to promote equitable healthcare to the LGBTQ older adult population and calls to attention the need for long-term care settings to uniformly follow best-practices.

